# Application of the multiple dependent state sampling strategy to late adolescent suicide rates

**DOI:** 10.1186/s12874-023-02007-2

**Published:** 2023-08-22

**Authors:** Nagasaritha Kolli, Kanaparthi Rosaiah, Gadde Srinivasa Rao, Peter Josephat Kirigiti

**Affiliations:** 1 Department of Community Medicine, NRI Medical College and Research Institute, Mangalagiri, Guntur India; 2 Department of Statistics, Acharya Nagarjuna University, Guntur, 522510 AP India; 3 Department of Mathematics and Statistics, The University of Dodoma, P.O. Box: 259, Dodoma, Tanzania

**Keywords:** Multiple dependent state sampling plan, New Lomax Rayleigh distribution, Average sample number, Suicide rates of late adolescents, Health care quality indicators, Quality gap analysis in hospitals

## Abstract

A multiple dependent state sampling plan (MDSSP) is designed when the lifetimes of the variables follow New Lomax Rayleigh Distribution (NLRD). The decision to accept or reject a lot in the proposed methodology is based on the quality of the given present or previous lots. A binomial model-based operating characteristic curve (OC curve) for continuous lots of variables under similar settings in healthcare is used in finding the probability of acceptance, acceptance number, rejection number, and the number of preceding (succeeding) lots to consider. Time truncated life test based on the specified median of the NLRD is used in designing the current acceptance sampling plan. For specified values of the parameters of NLRD, quantile ratios, consumer’s risk and producer’s risk, average sampling number (ASN) and probability of acceptance of a lot are reported in tables. Real data on worldwide suicide rates of 15–19 years in the year 2019 from the World Health Organization (WHO) website is considered to illustrate this methodology. The minimum sample size required from the selected data to comment on worldwide suicide rates in late adolescents is explained with MDSSP. The results of the proposed acceptance sampling method are compared with the single-stage sampling plan.

**Supplementary Information:**

The online version contains supplementary material available at 10.1186/s12874-023-02007-2.

## Introduction

Quality in the manufacturing or the service sector is a determinant for attracting demand. Continuous inspection and improving quality in the healthcare sector gained significance for the benefit of the patients and service providers. In this sector, quality is in two ways health-related and hospitality related. The former is objective, and the latter is subjective, it is equally important to measure both in the healthcare setting for healthy clinical outcomes and the satisfaction of the customer. Measuring, monitoring and controlling are parts of health policy design. Many health indicators are the targets of sustainable development goals (SDGs). Reducing deaths due to suicides is one of the priorities of the WHO mental health action plan 2013–2030 and is also goal 3.4 of SDGs. This paper aims to introduce the use of MDSSP in estimating the required sample size in monitoring and commenting on worldwide health indicators.

Sampling plans specify parameters of the sampling process like the number of samples to inspect from a selected lot, average Sampling Number (ASN), acceptance criteria: acceptable quality level (AQL), limiting quality level (LQL), producer’s (service providers) risk and consumer’s risk. Acceptance sampling plan (ASP), a tool of statistical quality control (SQC) spread its application wings to the manufacturing and service industry after its first use in World War II. In the service sector, inspect a lot with minimum cost, communicate with management to necessitate action. Acceptance sampling plans operating procedure is designed differently in lot-by-lot inspections and continuous flow processes (services). In the first case, single sampling and double sampling plans are useful, for the second type two-stage sampling, chain sampling, and multiple state dependent sampling plans are suitable.

The most common phenomenon in life testing is truncating a life test after a predefined time $t_{0}$ and noting the number of non confirming members/items in the inspecting lot before the pre specified time.Acceptance sampling plans literature for different symmetric and skewed distributions under truncated life testing is structured and coherent. The first single acceptance sampling plan based on exponential lifetimes was introduced by [1]. Truncated life test sampling plans based on normal and log-normal distributions are designed by [2]. Average life based sampling plans for log-logistic distribution are given by [3]. Rayleigh distribution based time truncated acceptance sampling plans were discussed by [4]. ASP for Birnbaum-Saunders distribution based on median life is illustrated by [5]. Percentile based acceptance sampling plans are given by [6, 7] for Birnbaum-Saunders and Burr type-XII distribution. Al-Nasser et al. [8] discussed Power Lomax acceptance sampling based on truncated life testing. Group acceptance sampling plans for resubmitted lots, size-biased Lomax distribution and MDSSP for exponentiated log-logistic distribution are given by [9, 10] respectively. Acceptance sampling plans for new Weibull-Pareto distribution based on percentiles are given by [11].

## Application of statistical quality control tools in health care

Unlike the manufacturing industry service sector suffers from more variability in outcome. Especially in health care service, input variability is more due to inevitable biological variability among human beings. So it is a great challenge to the healthcare sector to deliver quality clinical outcomes. Health care sector started employing SQC tools way back in the early nineteenth century mentioned in [12] or additional information on the SQC techniques usefulness in managing quality indicators in hospitals refer to [12–14]. Callahan et al. [15] used control charts to analyze patient waiting times and time from registration to the physician’s first orders. Rachmania et al. [16] discussed the application of a lot acceptance sampling plan in screening women and concluded that this procedure is useful in deciding the quality level of the indicator. Clemente et al. [17] mentioned the use of statistical process control in the emergency medicine department studying door-to-reperfusion time of myocardial infarction patients. Ray et al. [18] presented a case study in Indonesia on the use of control charts in hospitals. Rao et al. [19] discussed the use of control charts usefulness in monitoring surgical site infections and comparing mortality rates in CABG surgery and other surgeries. Quality indicators and benchmarks for different ICUs are given by [20]. The use of a multiple-state dependent sampling plan in assessing COVID-19 mortality rate lots is discussed by [21]. Statistical process control use in economic analysis and safety of chemotherapy batches in hospital pharmacies is mentioned by [22]. After this literature review, it is observed that distribution-based control charts and acceptance sampling plans were not of much use in healthcare quality analysis. With this motivation, we developed the MDSSP based on NLRD and its application in analyzing quality gaps and acceptability of worldwide suicide rates of late adolescents in 2019.

## Multiple dependant state sampling plans (MDSSP)

Conditional sampling plans are suitable for continuous process monitoring and improvement; it is first introduced by [23]. Chain sampling and multiple dependent state sampling come under continuous sampling plans. Different variations in chain sampling plans were studied by [24]. In 1976, [25] introduce another sampling plan, named MDSSP belonging to the conditional sampling family. MDSSP for measurement data was discussed by [26]. Aslam et al. [27] designed novel multiple dependent state sampling plan based on process loss consideration. The Multiple dependent state repetitive group sampling plan for normally distributed quality characteristics is given by [28]. Rao et al. [29] gave novel control charts based on multiple dependent state sampling plans. Some recent works to mention on MDSSP are [9, 10, 30] developed MDSSP based on repetitive group sampling for exponentiated half-logistic distribution and exponentiated Weibull distribution respectively. Modified multiple dependent state sampling plan is introduced and its economic efficiency is studied by [31]. The MDSSP efficiency over other sampling plans in reducing ASN is given by [32].

In the following sections, median-based probability of failure formulas for NLRD distribution and MDSSP when quality (health) indicator variable follows NLRD is derived.

## New Lomax Rayleigh distribution

NLRD is a new T-X family distribution proposed by [33] considering Lomax distribution, a special case of Pareto Type-II and Rayleigh distribution, its suitability in estimating life time data was verified. Different generalizations of Lomax distribution are available in the literature to make it suitable for studying lifetime data. To estimate drilling machine lifetime, [34] used the Lomax-G generator to derive Lomax-Rayleigh distribution and applied it to drilling machine data. Different types of estimators of Pareto-Rayleigh distribution are given by [35]. Different traditional and heuristic methods of estimation for the Lomax-Rayleigh distribution generated by the Lomax-X generator are given by [36]. Rady et al. [37] used Power Lomax distribution and tested its applicability in estimating the mean remission times of bladder cancer patients. Bladder cancer remission times data is studied with Pareto Weibull distribution by [38].

Let T be a quality indicator following NLRD with parameters $\theta ,\lambda$ and $\sigma$ then the cumulative distribution function (CDF) and probability density function (PDF) of the variable T are given as follows:

1 $$G\left( {t;\;\theta ,\;\lambda ,\;\sigma } \right) = 1 - \left[ {1 + \frac{{t^{2} }}{{2\lambda \sigma^{2} }}} \right]^{ - \theta } {\text{ , t}} > 0,\;\theta ,\lambda ,\sigma > 0$$

2 $$g\left( {t;\;\theta ,\;\lambda ,\;\sigma } \right) = \frac{\theta t}{{\lambda \sigma^{2} }}\left[ {1 + \frac{{t^{2} }}{{2\lambda \sigma^{2} }}} \right]^{{ - \left( {\theta + 1} \right)}} ,{\text{ t}} > 0,\;\theta ,\lambda ,\sigma > 0$$

where $\lambda$ and $\sigma$ are the scale parameters and $\theta$ is the shape parameter.

The $q^{th}$ quantile of NLRD is

3 $$t_{q} = \sigma \left[ {2\lambda \left[ {\left[ {1 - q} \right]^{{\frac{ - 1}{\theta }}} - 1} \right]} \right]^{\frac{1}{2}}$$

Let $$\eta_{q} = \left[ {2\lambda \left[ {\left[ {1 - q} \right]^{{\frac{ - 1}{\theta }}} - 1} \right]} \right]^{\frac{1}{2}}$$, then $$t_{q} = \sigma \eta_{q} \Rightarrow \sigma = \frac{{t_{q} }}{{\eta_{q} }}$$.

For specified values of $\lambda = \lambda_{0} ,\theta = \theta_{0}$, $t_{q}$ is a function of the scale parameter $\sigma = \sigma_{0}$.

Therefore $$\sigma_{0} = \frac{{t_{q} }}{{\left[ {2\lambda \left[ {\left[ {1 - q} \right]^{{\frac{ - 1}{\theta }}} - 1} \right]} \right]^{\frac{1}{2}} }}$$.

Median of the NLRD distribution, 50^th^ percentile is

4 $$t_{0.5} = \sigma \left[ {2\lambda \left[ {\left[ {1 - 0.5} \right]^{{\frac{ - 1}{\theta }}} - 1} \right]} \right]^{\frac{1}{2}}$$

Let *p* be the probability of failure of the variable following NLRD with terminating time $t_{0}$ and truncating time $t_{q}$, then $p = F(t_{0} )$. Experiment termination time can be expressed as $t_{0} = kt_{{q_{0} }}$.

5 $$p = 1 - \left[ {1 + \frac{{t_{0}^{2} }}{{2\lambda \sigma^{2} }}} \right]^{ - \theta }$$

By substituting $t_{0} = kt_{{q_{0} }}$ and $\sigma_{0}$ values in Eq. (5)

6 $$p = 1 - \left[ {1 + \frac{{\left( {kt_{{q_{0} }} } \right)^{2} }}{{2\lambda \left( {\frac{{t_{q} }}{{\eta_{q} }}} \right)^{2} }}} \right]^{ - \theta } \Rightarrow 1 - \left[ {1 + \frac{1}{{2\lambda \left( {\frac{{t_{q} }}{{t_{{q_{0} }} }}} \right)^{2} }}\left( {k\eta_{q} } \right)^{2} } \right]^{ - \theta }$$

Here it is important to consider both consumer’s risk and the producer’s risk. Quantile ratio plays a significant role in decision making about lot acceptance. As per Producer’s angle of lot acceptance quantile ratio must be at least one. If ${{(t_{q} } \mathord{\left/ {\vphantom {{(t_{q} } {t_{{q_{0} }} }}} \right. \kern-0pt} {t_{{q_{0} }} }}) > 1$, then the probability ($p_{1}$) obtained is considered as AQL and the consumer wants this ratio must be almost the pre defined level of the consumer’s risk $\beta$. LQL is the probability of this ratio ${{(t_{q} } \mathord{\left/ {\vphantom {{(t_{q} } {t_{{q_{0} }} }}} \right. \kern-0pt} {t_{{q_{0} }} }}) = 1$ being one($p_{2}$). NLRD parameters can be obtained from previous data on mortality rates. MDSSP is designed by substituting estimated parametric values of NLRD at different quantile ratios, terminating ratios, consumer’s risks and producer’s risk; the average sample number required to sentence a lot of world suicide rates is estimated with the WHO fact sheet data on suicides in 2019.

## Operating procedure for NLRD based MDSSP

The operating procedure and designing methodology of MDSSP based on NLRD are described in detail in the subsequent subsections.

### Operating procedure

MDSSP consists of the following symbols and notations:

*N*- Lot size, *n*- sample size,

$P_{a} (p)$-Probability of acceptance for a given *p,*

$p_{1}$-Probability of rejecting lot that has acceptable quality level (AQL), related to producers risk.

$p_{2}$-Probability of acceptance of lot with limiting quality level (LQL), associated with consumer’s risk.

$\alpha$- Producers risk, $\beta$-Consumers risk.

ASN- Average Sample Number

$c_{1} ,c_{2}$- Unconditional and conditional acceptance numbers respectively.

*m*- Required number of past or future lots to accept the present lot.

*d-* Number of units failed before the terminating time $t_{0}$ in the sample selected from the lot under test.

Step-1 From the lot, select a sample of units with size *n*, and put all these *n* items under test till predefined time $t_{0}$.

Step-2 Observe *d,* the number of units that failed before the test terminating time $t_{0}$.

Step-3Compare *d* with unconditional and conditional acceptance number $c_{1} ,c_{2}$ and make a decision.

- If $d \le c_{1}$ accept the lot otherwise reject the lot.
- If $c_{1} < d \le c_{2}$, then consider the number of failures in the preceding lots before terminating $t_{0}$ and this number must be less than $c_{1}$ in all the m previous lots.

The operating characteristic (OC) function of MDSSP is given by

7 $$P_{a} (p) = P(d \le c_{1} ;n) + \left[ {P(d \le c_{2} ;n) - P(d \le c_{1} ;n)} \right]\left[ {P(d \le c_{1} ;n} \right]^{m}$$

The probabilities in Eq. (7) are obtained by using the binominal theorem and the decision rule designed about lot acceptance at *p* is as follows:

8 $$P_{a} (p) = \sum\limits_{d = 0}^{{c_{1} }} {\left( \begin{gathered} n \hfill \\ d \hfill \\ \end{gathered} \right)} p^{d} \left( {1 - p} \right)^{n - d} + \sum\limits_{{d = c_{1} + 1}}^{{c_{2} }} {\left( \begin{gathered} n \hfill \\ d \hfill \\ \end{gathered} \right)p^{d} } \left( {1 - p} \right)^{n - d} \left( {\sum\limits_{d = 0}^{{c_{1} }} {\left( \begin{gathered} n \hfill \\ d \hfill \\ \end{gathered} \right)p^{d} \left( {1 - p} \right)^{n - d} } } \right)^{m}$$

### Designing methodology

The objective of designing any sampling plan is to avoid laborious 100% inspection and achieve ASN that minimizes the resources of the organizations. MDSSP also reduces the inspection time and cost by deciding the minimum number of units required to verify whether the sampled lot of quality indicator is acceptable or not. If the lot is rejected communication should be given to the management about the process (service). In this section, we tried to achieve minimum ASN through an optimization problem. The proposed optimization procedure for MDSSP based on NLRD is given below:

Minimize $$ASN(p) = n$$,

Subject to

$$P_{a} \left( {p_{1} } \right) \ge 1 - \alpha$$

$$P_{a} \left( {p_{2} } \right) \le \beta$$,

9 $$n > 1,m \ge 1,c_{2} > c_{1} \ge 0$$

The probability of AQL and LQL is obtained from the following equations:

10 $$P_{a} (p_{1} ) = \sum\limits_{d = 0}^{{c_{1} }} {\left( \begin{gathered} n \hfill \\ d \hfill \\ \end{gathered} \right)} p_{1}^{d} \left( {1 - p_{1} } \right)^{n - d} + \sum\limits_{{d = c_{1} + 1}}^{{c_{2} }} {\left( \begin{gathered} n \hfill \\ d \hfill \\ \end{gathered} \right)p_{1}^{d} } \left( {1 - p_{1} } \right)^{n - d} \left( {\sum\limits_{d = 0}^{{c_{1} }} {\left( \begin{gathered} n \hfill \\ d \hfill \\ \end{gathered} \right)p_{1}^{d} \left( {1 - p_{1} } \right)^{n - d} } } \right)^{m}$$

11 $$P_{a} (p_{2} ) = \sum\limits_{d = 0}^{{c_{1} }} {\left( \begin{gathered} n \hfill \\ d \hfill \\ \end{gathered} \right)} p_{2}^{d} \left( {1 - p_{2} } \right)^{n - d} + \sum\limits_{{d = c_{1} + 1}}^{{c_{2} }} {\left( \begin{gathered} n \hfill \\ d \hfill \\ \end{gathered} \right)p_{2}^{d} } \left( {1 - p_{2} } \right)^{n - d} \left( {\sum\limits_{d = 0}^{{c_{1} }} {\left( \begin{gathered} n \hfill \\ d \hfill \\ \end{gathered} \right)p_{2}^{d} \left( {1 - p_{2} } \right)^{n - d} } } \right)^{m}$$

In this paper median quantile ratio $t_{q} /t_{{q_{0} }}$ at the consumer’s risk must be at least 1, $t_{q} /t_{{q_{0} }} = 2,4,6,8,10$ are considered at the producer’s risk. The optimal parameters of the proposed plan for NLRD $\left( {\theta ,\lambda } \right) = \left( {1.5,1.5} \right),\left( {0.5,0.5} \right),\left( {1.5,1.0} \right),\left( {1.5,2.0} \right)$ are presented in the Tables 1, 2, 3 and 4. Values assumed for consumer’s risk are $\beta = 0.25,0.10,0.05,0.01$ and producer’s risk is considered as $\alpha = 0.05$ at 50^th^ percentile. The values considered for the termination ratio are $k = 0.5,0.7,1.0$. The computational work is done by using R software and R code is provided in Supplementary file.

**Table 1:** Optimal parameters of the proposed MDSSP for NLRD with $\theta = 1.5,\lambda = 1.5$

$\beta$	${{t_{q} } \mathord{\left/ {\vphantom {{t_{q} } {t_{{q_{0} }} }}} \right. \kern-0pt} {t_{{q_{0} }} }}$	$k = 0.5$					$k = 0.7$					$k = 1.0$				
		$n$	$c_{1}$	$c_{2}$	$m$	$p_{a} \left( {p_{1} } \right)$	$n$	$c_{1}$	$c_{2}$	$m$	$p_{a} \left( {p_{1} } \right)$	$n$	$c_{1}$	$c_{2}$	$m$	$p_{a} \left( {p_{1} } \right)$
0.25	2	21	2	4	2	0.9792	12	2	5	2	0.9774	7	2	3	2	0.9511
	4	7	0	6	3	0.9771	4	0	1	2	0.9772	3	0	2	1	0.9744
	6	7	0	6	3	0.9949	4	0	1	2	0.9949	3	0	2	1	0.9950
	8	7	0	6	3	0.9983	4	0	1	2	0.9983	3	0	2	1	0.9983
	10	7	0	6	3	0.9993	4	0	1	2	0.9993	3	0	2	1	0.9993
0.1	2	30	2	5	1	0.9532	20	3	13	2	0.9687	12	3	5	1	0.9601
	4	12	0	10	2	0.9574	7	0	1	1	0.9597	7	1	3	1	0.9973
	6	12	0	10	2	0.9903	7	0	1	1	0.9909	4	0	1	1	0.9884
	8	12	0	10	2	0.9967	7	0	1	1	0.9970	4	0	1	1	0.9961
	10	12	0	10	2	0.9986	7	0	1	1	0.9987	4	0	1	1	0.9983
0.05	2	40	3	13	2	0.9541	24	3	8	1	0.9560	16	4	6	1	0.9588
	4	17	0	3	1	0.9567	9	0	2	1	0.9531	8	1	3	1	0.9956
	6	15	0	10	2	0.9853	8	0	3	2	0.9841	5	0	2	1	0.9868
	8	15	0	10	2	0.9950	8	0	3	2	0.9946	5	0	2	1	0.9955
	10	15	0	10	2	0.9979	8	0	3	2	0.9977	5	0	2	1	0.9981
0.01	2	60	4	8	1	0.9535	38	5	9	1	0.9688	25	6	11	1	0.9711
	4	33	1	2	1	0.9851	19	1	4	1	0.9920	11	1	5	1	0.9876
	6	23	0	1	1	0.9753	13	0	3	1	0.9793	7	0	2	1	0.9751
	8	23	0	1	1	0.9915	13	0	3	1	0.9929	7	0	2	1	0.9915
	10	23	0	1	1	0.9964	13	0	3	1	0.9970	7	0	2	1	0.9964

**Table 2:** Optimal parameters of the proposed MDSSP for NLRD with $\theta = 0.5,\lambda = 0.5$

$\beta$	${{t_{q} } \mathord{\left/ {\vphantom {{t_{q} } {t_{{q_{0} }} }}} \right. \kern-0pt} {t_{{q_{0} }} }}$	$k = 0.5$					$k = 0.7$					$k = 1.0$				
		$n$	$c_{1}$	$c_{2}$	$m$	$p_{a} \left( {p_{1} } \right)$	$n$	$c_{1}$	$c_{2}$	$m$	$p_{a} \left( {p_{1} } \right)$	$n$	$c_{1}$	$c_{2}$	$m$	$p_{a} \left( {p_{1} } \right)$
0.25	2	16	2	12	2	0.9636	13	3	12	4	0.9611	12	4	11	2	0.9606
	4	5	0	4	4	0.9602	4	0	1	1	0.9652	5	1	2	1	0.9920
	6	5	0	4	4	0.9906	4	0	1	1	0.9919	3	0	2	1	0.9871
	8	5	0	4	4	0.9968	4	0	1	1	0.9973	3	0	2	1	0.9955
	10	5	0	4	4	0.9986	4	0	1	1	0.9988	3	0	2	1	0.9981
0.1	2	26	3	13	2	0.9519	20	4	7	2	0.9533	19	6	16	2	0.9534
	4	9	0	1	1	0.9517	10	1	2	1	0.9885	7	1	3	1	0.9872
	6	9	0	1	1	0.9888	6	0	1	1	0.9822	4	0	1	1	0.9706
	8	9	0	1	1	0.9962	6	0	1	1	0.9939	4	0	1	1	0.9896
	10	9	0	1	1	0.9984	6	0	1	1	0.9974	4	0	1	1	0.9955
0.05	2	35	4	10	2	0.9543	27	5	8	1	0.9558	24	7	12	1	0.9533
	4	18	1	2	1	0.9893	12	1	3	1	0.9904	8	1	3	1	0.9793
	6	11	0	10	2	0.9782	7	0	1	1	0.9762	5	0	2	1	0.9666
	8	11	0	10	2	0.9925	7	0	1	1	0.9917	5	0	2	1	0.9881
	10	11	0	10	2	0.9968	7	0	1	1	0.9964	5	0	2	1	0.9948
0.01	2	56	6	10	1	0.9648	40	7	12	1	0.9512	38	11	18	1	0.9605
	4	25	1	3	1	0.9860	16	1	4	1	0.9773	14	2	6	1	0.9894
	6	17	0	1	1	0.9633	11	0	3	1	0.9609	11	1	5	1	0.9955
	8	17	0	1	1	0.9871	11	0	3	1	0.9862	7	0	2	1	0.9777
	10	17	0	1	1	0.9945	11	0	3	1	0.9940	7	0	2	1	0.9902

**Table 3:** Optimal parameters of the proposed MDSSP for NLRD with $\theta = 1.5,\lambda = 1.0$

$\beta$	${{t_{q} } \mathord{\left/ {\vphantom {{t_{q} } {t_{{q_{0} }} }}} \right. \kern-0pt} {t_{{q_{0} }} }}$	$k = 0.5$					$k = 0.7$					$k = 1.0$				
		$n$	$c_{1}$	$c_{2}$	$m$	$p_{a} \left( {p_{1} } \right)$	$n$	$c_{1}$	$c_{2}$	$m$	$p_{a} \left( {p_{1} } \right)$	$n$	$c_{1}$	$c_{2}$	$m$	$p_{a} \left( {p_{1} } \right)$
0.25	2	21	2	4	2	0.9792	12	2	5	2	0.9774	7	2	3	2	0.9511
	4	7	0	6	3	0.9771	4	0	1	2	0.9772	3	0	2	1	0.9774
	6	7	0	6	3	0.9949	4	0	1	2	0.9949	3	0	2	1	0.9950
	8	7	0	6	3	0.9983	4	0	1	2	0.9983	3	0	2	1	0.9983
	10	7	0	6	3	0.9993	4	0	1	2	0.9993	3	0	2	1	0.9993
0.1	2	30	2	5	1	0.9532	20	3	13	2	0.9687	12	3	5	1	0.9601
	4	12	0	10	2	0.9574	7	0	1	1	0.9595	7	1	3	1	0.9973
	6	12	0	10	2	0.9903	7	0	1	1	0.9909	4	0	1	1	0.9884
	8	12	0	10	2	0.9967	7	0	1	1	0.997	4	0	1	1	0.9961
	10	12	0	10	2	0.9986	7	0	1	1	0.9987	4	0	1	1	0.9983
0.05	2	40	3	13	2	0.9541	24	3	8	1	0.956	16	4	6	1	0.9588
	4	17	0	3	1	0.9567	9	0	2	1	0.9531	8	1	3	1	0.9956
	6	15	0	10	2	0.9853	8	0	3	2	0.9841	5	0	2	1	0.9868
	8	15	0	10	2	0.995	8	0	3	2	0.9946	5	0	2	1	0.9955
	10	15	0	10	2	0.9979	8	0	3	2	0.9977	5	0	2	1	0.9981
0.01	2	60	4	8	1	0.9535	38	5	9	1	0.9688	25	6	11	1	0.9711
	4	33	1	2	1	0.9851	19	1	4	1	0.992	11	1	5	1	0.9876
	6	23	0	1	1	0.9753	13	0	3	1	0.9793	7	0	2	1	0.9751
	8	23	0	1	1	0.9915	13	0	3	1	0.9929	7	0	2	1	0.9915
	10	23	0	1	1	0.9774	13	0	3	1	0.997	7	0	2	1	0.9964

**Table 4:** Optimal parameters of the proposed MDSSP for NLRD with $\theta = 1.5,\lambda = 2.0$

$\beta$	${{t_{q} } \mathord{\left/ {\vphantom {{t_{q} } {t_{{q_{0} }} }}} \right. \kern-0pt} {t_{{q_{0} }} }}$	$k = 0.5$					$k = 0.7$					$k = 1.0$				
		*n*	*c*_1_	*c*_2_	*m*	*P*_*a*_*(p*_*1*_*) *	*n*	*c*_1_	*c*_2_	*m*	*P*_*a*_*(p*_*1*_*) *	*n*	*c*_1_	*c*_2_	*m*	*P*_*a*_*(p*_*1*_*) *
0.25	2	21	2	4	2	0.9792	12	2	5	2	0.9774	7	2	3	2	0.9511
	4	7	0	6	3	0.9771	4	0	1	2	0.9772	3	0	2	1	0.9774
	6	7	0	6	3	0.9949	4	0	1	2	0.9949	3	0	2	1	0.995
	8	7	0	6	3	0.9983	4	0	1	2	0.9983	3	0	2	1	0.9983
	10	7	0	6	3	0.9993	4	0	1	2	0.9993	3	0	2	1	0.9993
0.1	2	30	2	5	1	0.9532	20	13	13	2	0.9687	3	0	5	1	0.9601
	4	12	0	10	2	0.9574	7	1	1	1	0.9597	12	3	3	1	0.9973
	6	12	0	10	2	0.9903	7	1	1	1	0.9909	7	1	1	1	0.9884
	8	12	0	10	2	0.9967	7	1	1	1	0.9970	4	0	1	1	0.9961
	10	12	0	10	2	0.9986	7	1	1	1	0.9987	4	0	1	1	0.9983
0.05	2	40	3	13	2	0.9541	24	8	8	1	0.9560	4	0	6	1	0.9588
	4	17	0	3	1	0.9567	9	2	2	1	0.9531	16	4	3	1	0.9956
	6	15	0	10	2	0.9853	8	3	3	2	0.9841	8	1	2	1	0.9868
	8	15	0	10	2	0.9950	8	3	3	2	0.9946	5	0	2	1	0.9955
	10	15	0	10	2	0.9979	8	3	3	2	0.9977	5	0	2	1	0.9981
0.1	2	60	4	8	1	0.9535	38	9	9	1	0.9688	5	0	11	1	0.9711
	4	33	1	2	1	0.9851	19	4	4	1	0.9920	25	6	5	1	0.9876
	6	23	0	1	1	0.9753	13	3	3	1	0.9793	11	1	2	1	0.9751
	8	23	0	1	1	0.9915	13	3	3	1	0.9929	7	0	2	1	0.9915
	10	23	0	1	1	0.9964	13	3	3	1	0.9970	7	0	2	1	0.9964

Results observed from Tables 1, 2, 3 and 4 when parametric combinations are fixed are as follows.

1. An inverse relationship is observed between sample size and consumer’s risk. In all the Tables 1, 2, 3 and 4 sample size increases when the consumer’s risk decreases.
2. Sample size decreases as the termination ratio $k$ increases from 0.5 to 1.0
3. Probability of lot acceptance increases along with the quantile ratio. As $t_{q} /t_{{q_{0} }}$ approaches ‘10’ probability of lot acceptance also increases and approximates to almost ‘1’.

## Applications of MDSSP for real data

In this subsection, we demonstrate the proposed methodology with two real data sets one related to public health issue and engineering application related to failure time of the components.

### Worldwide suicide rates data

Suicide is a preventable death. As it is a global public health issue impacting all age groups, suicide prevention is a high priority condition in the WHO Mental Health Action Plan 2013–2030. Losing an adolescent in suicide is a great loss to the family as well as the country. Reasons for suicide in middle and late adolescence may be due to not addressing mental health issues. The adolescent state is more vulnerable to mental health problems due to psycho-physiological changes, environment and circumstances. Late adolescence in India has become stressful due to their senior secondary school education under heavy pressure towards productivity and achievement without any physical activity. According to WHO Facts on Suicide 2019, suicide is the fourth leading cause of death among late adolescents. The major contribution (77%) of global suicidal deaths is from low- and middle income countries (data available in Supplementary Material file).

As per the WHO fact sheet on 15–19 years suicides in the year 2019, overall suicide rates are high in Western specific, Europe and American countries (Guyana (40.37 per 100,000)) in India it is reported as 10.4 per 100,000. In females highest percentage of suicides is reported in the American country Guyana (45.71 per 100,000), Indian female late adolescents suicide rate is 14.55 per 100,000 which is double that of males (6.71 per 100,000). The highest suicide rate in males was reported in the Western Pacific country, Kiribati (51.93 per 100,000). Worldwide, it is the need of the hour to design effective suicide prevention strategies to reduce its contribution to premature deaths due to non- communicable diseases. From the above discussion it is observed that, world suicide rates of late adolescents’ data are highly skewed, the applicability of NLRD distribution proposed by [33] to heavily right-skewed data is verified and compared with some other existing distributions.

**Fig. 1:**
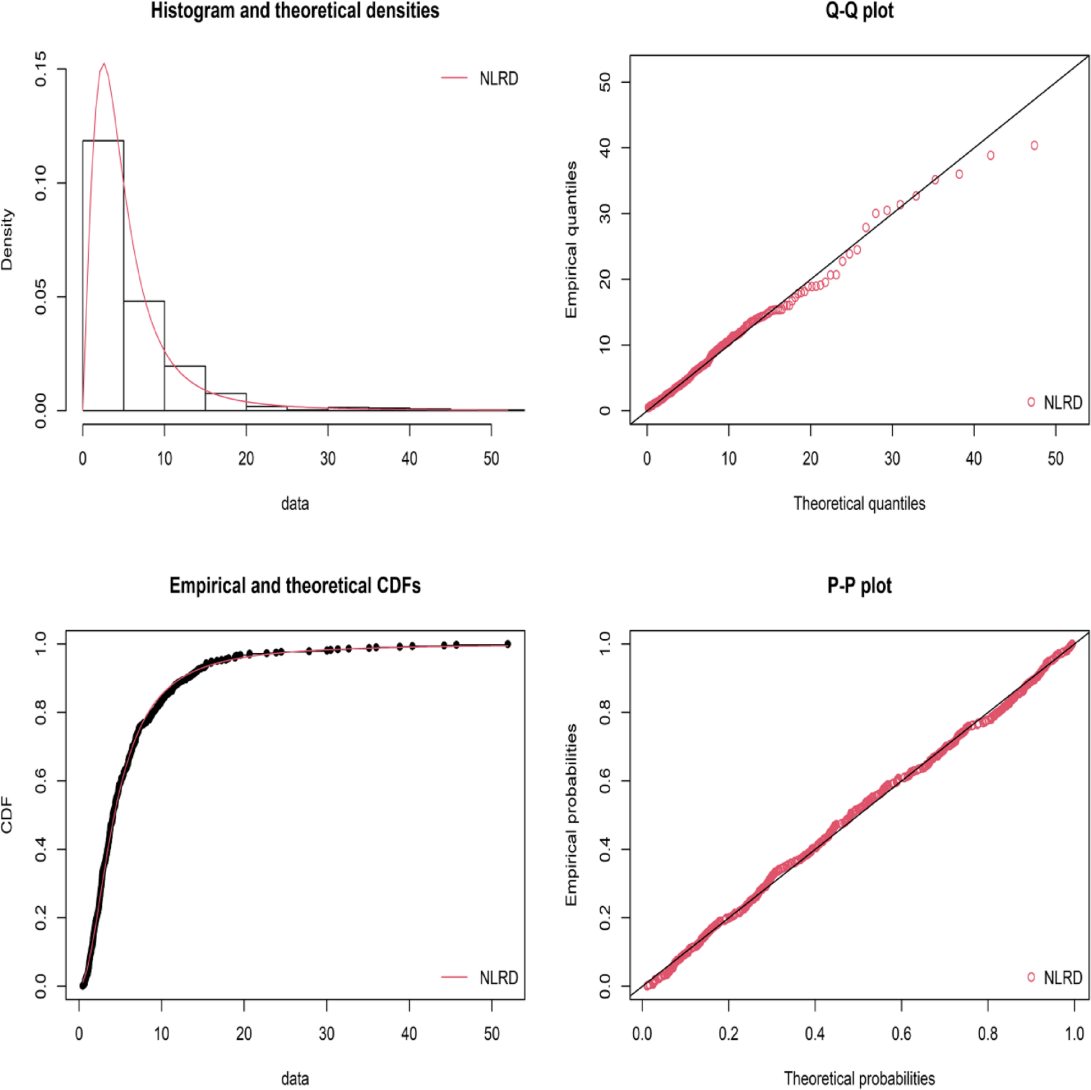
Visual presentation of fitted model for worldwide rates of suicide deaths data

In this section worldwide suicide mortality rates downloaded from WHO factsheet data on suicides in 2019 in the age group 15–19 are used to estimate the parameters of the NLRD. The goodness of fit of the NLRD distribution to this data is tested by using the Kolmogorov–Smirnov test, the value of D is 0.0291 and the *p*- value is 0.7908. Estimated values of the parameters of the NLRD are obtained by using the maximum likelihood estimation method and they are $\widehat{\theta } = 1.0559$, $\widehat{\lambda } = 0.6867$ and $\hat{\sigma } = 3.7817$. Figure 1 displayed the visual presentation of fitted model, these graphs explain to us about the goodness of fit of the NLRD distribution to worldwide suicide rates of all the countries.

For instance, the industrialist would like to use the developed multiple dependent state sampling plans to implement the median life percentile of the product where the product lifetime follows NLRD with the shape parameters are $\widehat{\theta } = 1.0559$ and $\widehat{\lambda } = 0.6867$. By 2030, suicide prevention and reducing the suicide mortality rate by one third is Goal 3 of Sustainable Development Goals. It may be necessary to check whether the suicide death rates are reduced year by year or not. The medical practitioners suggest that the given median suicide rate is 0.95 whereas the medical practitioners expected that the median suicide rate is 1.9. The consumer’s risk is 0.05 if the actual median suicide rate is 0.95 and the producer’s risk is 0.10 if the actual median suicide rate is 1.9. With these constraints, the optimal parameters selected from Table 5 are *n* = 37, $c_{1}$ = 3, $c_{2}$ = 9, and *m* = 2 with values of $\widehat{\theta } = 1.0559$, $\widehat{\lambda } = 0.6867$,$t_{{q_{0} }}$ = 0.75, $\alpha$ = 0.05, $\beta$ = 0.05, $t_{q} /t_{{q_{0} }}$ = 2 at *k* = 0.5. The MDSSP are illustrated as follows:

**Table 5:** Optimal parameters of the proposed MDSSP for NLRD with $\hat{\theta } = 1.056,\hat{\lambda } = 0.6867$

$\beta$	${{t_{q} } \mathord{\left/ {\vphantom {{t_{q} } {t_{{q_{0} }} }}} \right. \kern-0pt} {t_{{q_{0} }} }}$	$k = 0.5$					$k = 0.7$					$k = 1.0$				
		$n$	$c_{1}$	$c_{2}$	$m$	$p_{a} \left( {p_{1} } \right)$	$n$	$c_{1}$	$c_{2}$	$m$	$p_{a} \left( {p_{1} } \right)$	$n$	$c_{1}$	$c_{2}$	$m$	$p_{a} \left( {p_{1} } \right)$
0.25	2	19	2	3	3	0.9601	11	2	10	5	0.9545	8	2	5	1	0.9598
	4	7	0	6	2	0.9807	4	0	3	2	0.9765	3	0	2	1	0.9731
	6	7	0	6	2	0.9958	4	0	3	2	0.9948	3	0	2	1	0.9940
	8	7	0	6	2	0.9986	4	0	3	2	0.9983	3	0	2	1	0.9980
	10	7	0	6	2	0.9994	4	0	3	2	0.9993	3	0	2	1	0.9992
0.1	2	32	3	13	3	0.9670	19	3	13	2	0.9636	14	4	13	2	0.9707
	4	11	0	10	2	0.9562	6	0	2	2	0.9511	7	1	3	1	0.9963
	6	11	0	10	2	0.9900	6	0	2	2	0.9887	4	0	1	1	0.9860
	8	11	0	10	2	0.9967	6	0	2	2	0.9962	4	0	1	1	0.9953
	10	11	0	10	2	0.9986	6	0	2	2	0.9984	4	0	1	1	0.9980
0.05	2	37	3	9	2	0.9508	26	4	6	1	0.9655	18	5	10	2	0.9695
	4	16	0	3	1	0.9533	13	1	2	1	0.9917	8	1	3	1	0.9939
	6	14	0	10	2	0.9843	8	0	1	1	0.9857	5	0	2	1	0.9840
	8	14	0	10	2	0.9947	8	0	1	1	0.9952	5	0	2	1	0.9946
	10	14	0	10	2	0.9978	8	0	1	1	0.9980	5	0	2	1	0.9977
0.01	2	63	5	8	1	0.9673	37	5	10	1	0.9583	25	6	11	1	0.9543
	4	31	1	2	1	0.9833	18	1	4	1	0.9907	11	1	5	1	0.9831
	6	21	0	10	2	0.9671	12	0	1	1	0.9694	7	0	2	1	0.9701
	8	21	0	10	2	0.9886	12	0	1	1	0.9895	7	0	2	1	0.9897
	10	21	0	10	2	0.9951	12	0	1	1	0.9955	7	0	2	1	0.9956

In this context, a lot is a group of suicide rates of some of the countries selected at random from worldwide suicide rates. For example, a sample of 37 countries suicide rates of late adolescents in the age group of 15–19 years will be selected at random for the group of young people and check their suicide rate is 0.95. If the suicide rate before 0.95 is in 3 countries in a group of the population will be accepted and the group of the population will be rejected if it is greater than 9 countries in a group. There will be indecision of the group of the population is deferred until the 2 preceding the group of the population will be tested in case of the suicide rate of countries in younger people in a group of the population is between 3 and 9. For this real example, there are 16 countries’ suicide rates in younger people in a group of the population before the suicide rate before 0.75. Hence, reject the suicide rates of the countries in late adolescent people in a group of the population. Thus medical practitioners could suggest to the government or public that the median suicide rate of countries in late adolescent people in a group of the population is at an unacceptable level.

Performance of NLRD is compared with five other related distributions; Rayleigh distribution (RD), Lomax distribution (LD), Pareto Type–II(PD2), Power Lomax distribution (PLD) and Lomax Rayleigh distribution (LRD). Estimates of -2log likelihood, AIC, BIC, CAIC and HQIC of NLRD compared with other above mentioned distributions is given below. A better fit of the distributions to real data sets is estimated with Cramer-Von Mises statistic (W^*^), Anderson–Darling statistic (A^*^) and Kolmogrov-Smirnov statistic (K-S).The distribution with lower A,W, and K-S values is a better fit. Maximum likelihood estimates of parameters of the NLRD and K-S test p-values are presented in Table 6 and AIC, BIC, CAIC, HQIC, W^*^ and A^*^ values are reported in Table 7. From Tables 6 and 7 it is evident that, the intended model is a satisfactory model among several models. The Performance of NLRD is compared with five other related distributions RD, LD, PD2, PLD and LRD. In both real data sets, our anticipated model fits perfectly when compared to all other distributions. As a result, the anticipated model is an alternative model for real data modeling in lifetime data, specifically in the medical field.

**Table 6:** Estimate of the parameters, K-S statistic and *p*-value for worldwide suicide data set

Model	Estimate (SE)			K-S D Value	*P*-Value
**NLRD ** $(\theta ,\lambda ,\sigma )$	**1.0559 (0.0968)**	**0.6866 (0.0074)**	**3.7817 (6.1241)**	**0.0291**	**0.7908**
RD ($\sigma$)	6.32 (0.1369)			0.3324	2.22e-16
LD ($\theta ,\lambda$)	41.7221 (60.1165)	252.1388 (371.8549)		0.1268	7.201e-08
PD2 ($\alpha ,\sigma$)	85.4733 (77.8912)	522.9975 (652.1842)		0.1251	1.1243e-07
PLD $(\alpha ,\beta ,\lambda )$	0.9988 (0.2482)	(2.2196) (0.2153)	26.5636(5.1004)	0.0563	0.0679
LRD $(\alpha ,\theta )$	1.0777 (0.0968)	0.0493 (0.0074)		0.0295	0.7396

**Table 7:** The AIC, BIC, CAIC, HQIC, W^*^ and A^*^ values of distributions for worldwide suicide data

Model	-2*ll*	AIC	CAIC	BIC	HQIC	W^*^	A^*^
**NLRD ** $(\theta ,\lambda ,\sigma )$	**1445.4717**	**2896.9434**	**2896.9887**	**2909.7789**	**2901.9662**	**0.1441**	**0.9350**
RD ($\sigma$)	1720.9616	3443.9232	3443.9307	3448.2017	3445.5975	2.7095	16.0523
LD($\theta ,\lambda$)	1504.5923	3013.1846	3013.2072	3021.7416	3016.5331	0.9125	5.4559
PD($\alpha ,\sigma$)	1504.6856	3013.3171	3013.3398	3021.8742	3016.6627	0.9520	5.6965
PLD $(\alpha ,\beta ,\lambda )$	1446.9579	2899.9611	2899.9611	2912.7513	2904.9306	0.1563	1.0268
LRD $(\alpha ,\theta )$	1445.4963	2894.9925	2895.0152	2903.5436	2898.3411	0.1508	0.9717

### Failure time data

We consider the real time application to illustrate the suggested MDS sampling plan for engineering industrial use, if lifetime of an item comes from NLRD with unknown shape parameters. The following data is acquired from [39] and this data set corresponds to time between failures of 30 repairable components. Failure time (days): 0.11, 0.30, 0.40, 0.45, 0.59, 0.63, 0.70, 0.71, 0.74, 0.77, 0.94, 1.06, 1.17, 1.23, 1.23, 1.24, 1.43, 1.46, 1.49, 1.74, 1.82, 1.86, 1.97, 2.23, 2.37, 2.46, 2.63, 3.46, 4.36, 4.73.

**Fig. 2:**
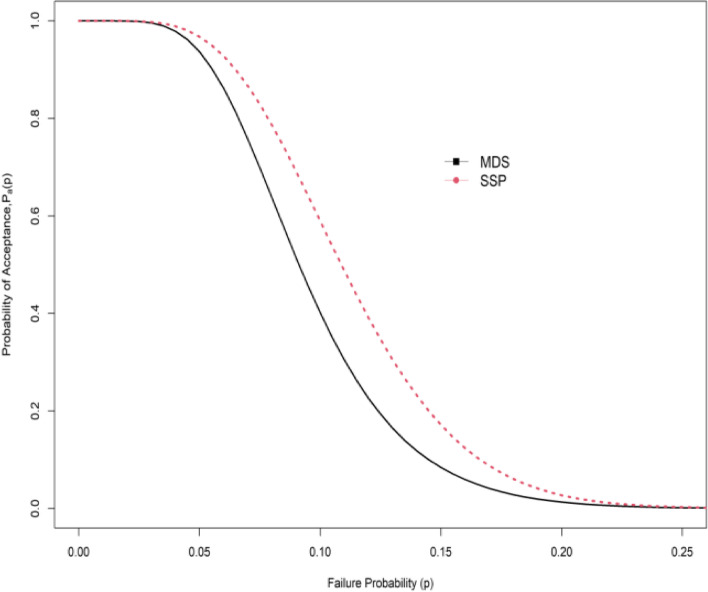
Visual presentation of fitted model for failure time data

The demonstration of the goodness of fit for the given model is shown in Fig. 2, the empirical and theoretical cdfs and Q-Q plots for the NLRD for the time between failures of repairable items and also in Fig. 2, Estimates of the density functions for the time between failures of repairable items. The maximum likelihood estimation of the parameters of NLRD for the time between failures of 30 repairable items is are $\hat{\theta }$ = 1.9273 and $\hat{\lambda }$ = 1.8958 and the maximum distance between the real time data and the fitted of NLRD was found from the Kolmogorov–Smirnov test is 0.0862 and also the *p*-value is 0.979.

Assume that the investigator wish for implement the median life of the product for the proposed MDS sampling scheme when the product lifetime comes from NLRD with estimated shape parameter is $\hat{\theta }$ = 1.9273 and $\hat{\lambda }$ = 1.8958. Let the specified quantile time between failures of repairable items is 0.5 i.e. $t_{q}^{0} = 0.65$ and the experiment termination time is 0.65, i. e. $t_{0}$ = 0.65. Hence, the termination constant, $k = {{t_{0} } \mathord{\left/ {\vphantom {{t_{0} } {t_{q}^{0} }}} \right. \kern-0pt} {t_{q}^{0} }} = 1.0$. Assuming that,$\alpha = 0.05$, $\beta = 0.10$,$k = 1.0$ and ${{t_{q} } \mathord{\left/ {\vphantom {{t_{q} } {t_{q}^{0} }}} \right. \kern-0pt} {t_{q}^{0} }}$ = 2, so from Table 8 the design parameters are *n* = 30, $c_{1}$ = 2, $c_{2}$ = 4 and *m* = 1. Thus, the design could be carried out as persists: obtain 30 samples at random from the current lot. Accept the lot if two failures occur before 0.65 time between failures of repairable items. If more than 4 failures the lot should be rejected. Whereas, if the number of failures are between 2 and 4, the outlook of the lot will be dependent until the preceding one lot is tested. If the preceding one lot is accepted, then product could be accepted. Otherwise, reject present lot. For the present example six failures are noted before the time between failures of repairable items 0.65. Therefore, the lot is rejected.

**Table 8:** Optimal parameters of the proposed MDSSP for NLRD with $\hat{\theta } = 1.9273,\hat{\lambda } = 1.8958$

$\beta$	${{t_{q} } \mathord{\left/ {\vphantom {{t_{q} } {t_{{q_{0} }} }}} \right. \kern-0pt} {t_{{q_{0} }} }}$	$k = 0.5$					$k = 0.7$					$k = 1.0$				
		$n$	$c_{1}$	$c_{2}$	$m$	$p_{a} \left( {p_{1} } \right)$	$n$	$c_{1}$	$c_{2}$	$m$	$p_{a} \left( {p_{1} } \right)$	$n$	$c_{1}$	$c_{2}$	$m$	$p_{a} \left( {p_{1} } \right)$
0.25	2	21	2	3	3	0.9653	12	2	3	2	0.9643	7	2	3	2	0.9571
	4	8	0	7	2	0.9815	4	0	1	2	0.9793	3	0	2	1	0.9794
	6	8	0	7	2	0.9960	4	0	1	2	0.9955	3	0	2	1	0.9955
	8	8	0	7	2	0.9987	4	0	1	2	0.9985	3	0	2	1	0.9985
	10	8	0	7	2	0.9995	4	0	1	2	0.9994	3	0	2	1	0.9994
0.10	2	30	2	4	1	0.9515	17	2	5	1	0.9514	12	3	5	1	0.9665
	4	12	0	3	2	0.9613	7	0	1	1	0.9634	4	0	1	1	0.9539
	6	12	0	3	2	0.9913	7	0	1	1	0.9918	4	0	1	1	0.9895
	8	12	0	3	2	0.9971	7	0	1	1	0.9973	4	0	1	1	0.9965
	10	12	0	3	2	0.9988	7	0	1	1	0.9989	4	0	1	1	0.9985
0.05	2	41	3	10	2	0.9592	24	3	6	1	0.9602	16	4	6	1	0.9661
	4	16	0	1	1	0.9504	9	0	2	1	0.9574	8	1	3	1	0.9963
	6	16	0	1	1	0.9887	9	0	2	1	0.9905	5	0	2	1	0.9881
	8	16	0	1	1	0.9963	9	0	2	1	0.9969	5	0	2	1	0.9960
	10	16	0	1	1	0.9984	9	0	2	1	0.9987	5	0	2	1	0.9983
0.01	2	62	4	8	1	0.9574	38	5	11	2	0.9603	22	5	8	1	0.9557
	4	35	1	3	1	0.9934	19	1	3	1	0.9924	11	1	5	1	0.9895
	6	24	0	1	1	0.9759	13	0	2	1	0.9809	7	0	2	1	0.9775
	8	24	0	1	1	0.9918	13	0	2	1	0.9936	7	0	2	1	0.9924
	10	24	0	1	1	0.9965	13	0	2	1	0.9973	7	0	2	1	0.9968

### Comparison of proposed MDSSP with the single-stage sampling plan

**Fig. 3:**
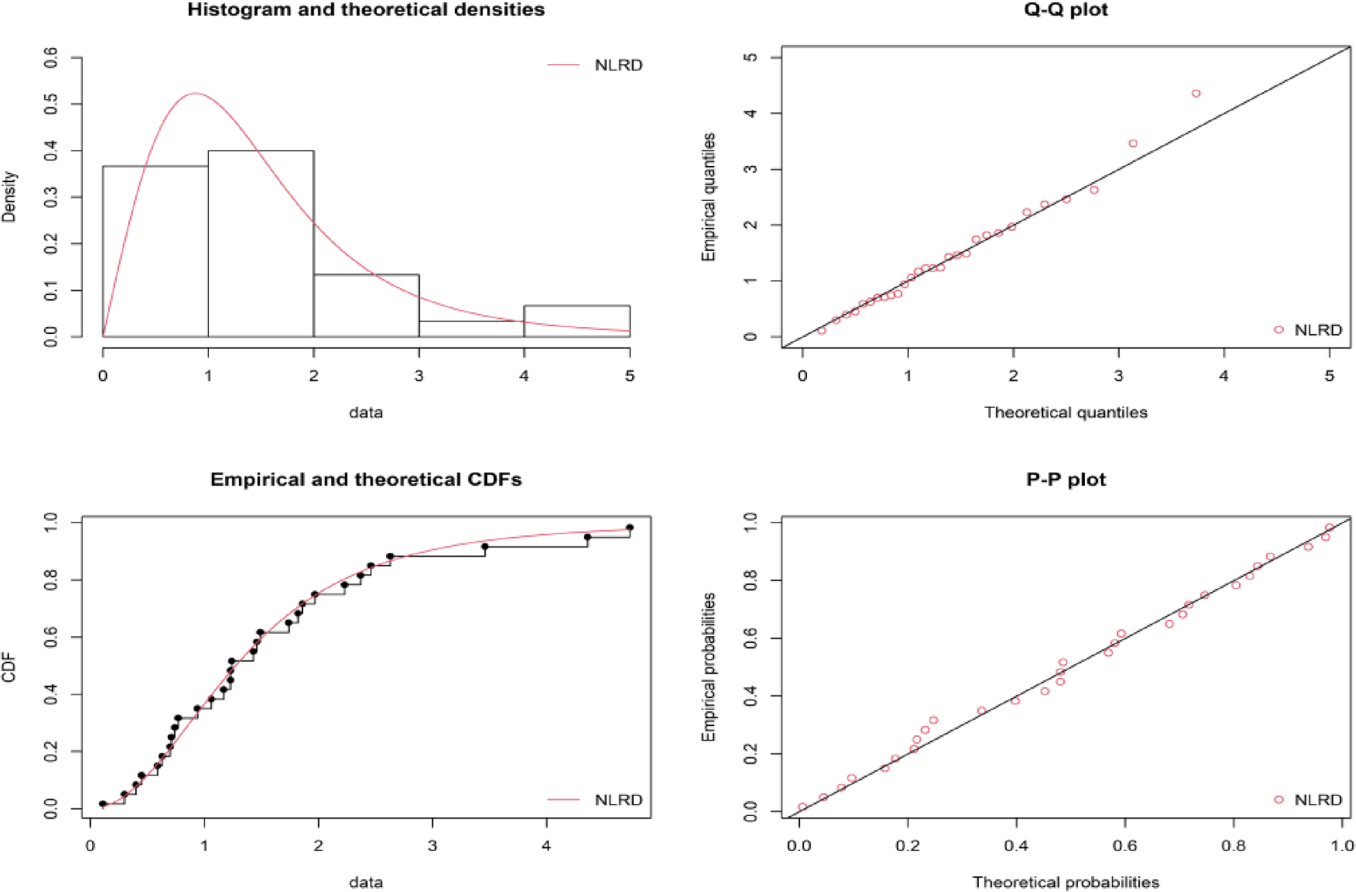
_Comparison OC curves for MDSSP and SSP_

The comparison is made for single and MDSSP when quality control follows NLRD, the OC curve is used here to show the efficiency of the plan. The curve has displayed the difference in probabilities of accepting a good lot as well as rejecting a bad lot. Table 9, has revealed the efficiency of the proposed MDSSP over the single sampling plan (SSP) while assuming the underlying distribution of data to follow NLRD. Considering the quantile ratio ${{t_{q} } \mathord{\left/ {\vphantom {{t_{q} } {t_{{q_{0} }} }}} \right. \kern-0pt} {t_{{q_{0} }} }} = 2,4,6,8,10$ for each consumer's risk $\beta = 0.25,0.10,0.05,0.01$ while keeping the producer’s risk at $\alpha = 0.05$. The comparison is basically on the sample size $n$ and probability of acceptance $P_{a} \left( {p_{1} } \right)$. The acceptance sample size for the proposed MDSSP is smaller than the existing single sampling plan for several set parameters see Table 9. For quantile ratio 2, the plan parameters for the MDSSP are* n* = 21, $c_{1}$ = 2, $c_{2}$ = 4, *m* = 2 whereas for SSP the design parameters are *n* = 33, c_1_= 4 with a corresponding probability of acceptances are 0.9792 and 0.9720 respectively when $\beta = 0.25$, *r* = 2, $\theta = 1.5, \, \lambda { = 1}{\text{.5}}$. The sample size is smaller for the MDSSP as compared with SSP. As the quantile ratio increased the acceptance sample size decreased for both sampling plans. Figure 3 depicts the OC curve for comparison of MDSSP with plan parameters *n* = 40, $c_{1}$ = 3, $c_{2}$ = 13, *m* = 2 and SSP with *n* = 61, $c_{1}$ = 6 when $\beta = 0.05$. It is noticed that the MDSSP is convincingly greater efficient than SSP in terms of sample size.

**Table 9:** Comparison of ASN values between MDSSP and SSP when $\hat{\theta } = 1.5,\hat{\lambda } = 1.5$

$\beta$	${{t_{q} } \mathord{\left/ {\vphantom {{t_{q} } {t_{{q_{0} }} }}} \right. \kern-0pt} {t_{{q_{0} }} }}$	$k = 0.5$								$k = 1.0k = 1.0$							
		MDSSP					SSP			MDSSP					SSP		
		$n$	$c_{1}$	$c_{2}$	$m$	$P_{a} (p_{1} )$	$n$	$c$	$P_{a} (p_{1} )$	*n*	$c_{1}$	$c_{2}$	$m$	$P_{a} (p_{1} )$	$n$	$c$	$P_{a} (p_{1} )$
0.25	2	21	2	4	2	0.9792	33	4	0.9720	7	2	3	2	0.9511	14	5	0.9685
	4	7	0	6	3	0.9771	14	1	0.9849	3	0	2	1	0.9744	5	1	0.9751
	6	7	0	6	3	0.9949	7	0	0.9582	3	0	2	1	0.995	2	0	0.9526
	8	7	0	6	3	0.9983	7	0	0.9762	3	0	2	1	0.9983	2	0	0.9730
	10	7	0	6	3	0.9993	7	0	0.9847	3	0	2	1	0.9993	2	0	0.9826
0.1	2	30	2	5	1	0.9532	48	5	0.9607	12	3	5	1	0.9601	19	6	0.9522
	4	12	0	10	2	0.9574	20	1	0.9701	7	1	3	1	0.9973	7	1	0.9512
	6	12	0	10	2	0.9903	20	1	0.9935	4	0	1	1	0.9884	7	1	0.9889
	8	12	0	10	2	0.9967	12	0	0.9596	4	0	1	1	0.9961	7	1	0.9963
	10	12	0	10	2	0.9986	12	0	0.9739	4	0	1	1	0.9983	4	0	0.9655
0.05	2	40	3	13	2	0.9541	61	6	0.9593	16	4	6	1	0.9588	23	7	0.9508
	4	17	0	3	1	0.9567	24	1	0.9581	8	1	3	1	0.9956	11	2	0.9825
	6	15	0	10	2	0.9853	24	1	0.9906	5	0	2	1	0.9868	8	1	0.9854
	8	15	0	10	2	0.9950	24	1	0.9969	5	0	2	1	0.9955	8	1	0.9951
	10	15	0	10	2	0.9979	15	0	0.9675	5	0	2	1	0.9981	5	0	0.9570
0.1	2	60	4	8	1	0.9535	89	8	0.9552	25	6	11	1	0.9711	35	10	0.9525
	4	33	1	2	1	0.9851	42	2	0.9805	11	1	5	1	0.9876	14	2	0.9657
	6	23	0	1	1	0.9753	33	1	0.9827	7	0	2	1	0.9751	11	1	0.9726
	8	23	0	1	1	0.9915	33	1	0.9942	7	0	2	1	0.9915	11	1	0.9906
	10	23	0	1	1	0.9964	23	0	0.9506	7	0	2	1	0.9964	11	1	0.9960

## Conclusions

Multiple dependent state sampling plans are developed when the lifetimes follow the New Lomax Rayleigh distribution. Lifetimes are truncated at a specified time point. At predefined producer’s and consumer’s risk optimal parameters of the proposed sampling plans are estimated. As suicide rates are continuously observed over years, and reducing suicide deaths is one of the SDGs, application of the proposed MDSSP to check acceptability of the countries’ suicide rates worldwide in late adolescents is verified when the data follows NLRD. We conclude that the proposed MDSSP is more efficient than the existing plans and it can be applied to verify the acceptability of different variables in health statistics. In future applications, multiple dependent state sampling plans may be applied in understanding health indicators quality, hospital equipment manufacturing industries can use this plan to sentence on manufactured lot quality (a lot of health quality indicators) and the average sampling number needs to decide on the quality of the lot. Some examples where it can be applied to evaluate the quality in the utilization of operation theatre timings, waiting times in outpatient departments, waiting times for lab reports and quality care in life-saving treatments like the door to balloon times in cardiology, etc. The proposed MDSSP helps to understand the quality of services provided today not only based on today’s data but also from previous days’ lots to ensure consistency in quality health care. The future scope of the work is to develop NLRD based attribute control charts, control charts based on multiple dependent state sampling, multiple dependent state repetitive sampling and modified multiple dependent state sampling plan for NLRD distributed quality indicators.

### Supplementary Information

**Additional file 1.**

## Data Availability

Data is available in Supplementary Material file. Source of the data link is also provided. The datasets generated and/or analysed during the current study are available in the [WHO website] repository, [who.int/data/gho/data/themes/mental-health/suicide-rates].

## Abbreviations

ASN: Average sampling number
AQL: Acceptable quality level
CDF: Cumulative distribution function
PDF: Probability density function
LQL: Limiting quality level
ASP: Acceptance sampling plan
MDSSP: Multiple dependent state sampling
NLRD: New Lomax Rayleigh Distribution
OC curve: Operating characteristic curve
SDGs: Sustainable development goals
SQC: Statistical quality control
WHO: World health organization

## References

[1] Epstein B (1954). Truncated Life tests in the exponential case. Ann Math Stat.

[2] Gupta SS (1962). Life test sampling plans for normal and lognormal distributions. Technometrics.

[3] Kantam RRL, Rosaiah K, Rao GS (2001). Acceptance sampling based on life tests: log-logistic model. J Appl Stat.

[4] Baklizi A, El-Masri A-Q, AL-Nasser A (2005). Acceptance sampling plans in the rayleigh model. Commun Stat Appl Methods.

[5] Balakrishnan N, Leiva V, López J (2007). Acceptance sampling plans from truncated life tests based on the generalized Birnbaum-Saunders distribution. Commun Stat Simul Comput.

[6] Lio YL, Tsai TR, Wu SJ (2009). Acceptance sampling plans from truncated life tests based on the birnbaum-saunders distribution for percentiles. Commun Stat Simul Comput.

[7] Lio YL, Tsai TR, Wu SJ (2010). Acceptance sampling plans from truncated life tests based on the Burr type XII percentiles. J Chinese Inst Ind Eng.

[8] Al-Nasser AD, ulHaq MA (2021). Acceptance sampling plans from a truncated life test based on the power Lomax distribution with application to manufacturing. Stat Transit.

[9] Rao GS, Rosaiah K, Rameshnaidu C (2020). Design of multiple-deferred state sampling plans for exponentiated half logistic distribution. Cogent Math Stat.

[10] Rao GS, Jilani SD, Rao AV (2021). Designing of multiple dependent state repetitive sampling plan for type-II generalized half logistic distribution. Int J Syst Assur Eng Manag.

[11] Shrahili M, Al-Omari AI, Alotaibi N (2021). Acceptance sampling plans from life tests based on percentiles of new weibull–pareto distribution with application to breaking stress of carbon fibers data. Processes.

[12] Benneyan JC (1998). Statistical quality control methods in infection control and hospital epidemiology, part II: chart use, statistical properties, and research issues. Infect Control Hosp Epidemiol.

[13] Finison LJ, Finison KS (1996). Applying control charts to quality improvement. J Healthc Qual.

[14] Jané AC, Cintas PG (1999). Lot sampling plans in the measure of quality of care indicators. Int J Qual Heal Care.

[15] Callahan CD, Griffen DL (2003). Advanced statistics: Applying statistical process control techniques to emergency medicine: a primer for providers. Acad Emerg Med.

[16] Rachmania I, Setyaningsih S, Rakhmaniar M, Basri M (2012). Application of quality tools and techniques in hospital: case study in Bandung Indonesia.

[17] Clemente F, Papi M, Pontecorvi L, Menichetti A (2016). Evaluation of indices for the measurement of quality in health systems. Int J Metrol Qual Eng.

[18] Ray B, Samaddar DP, Todi SK, Ramakrishnan N, John G, Ramasubban S (2009). Quality indicators for ICU: ISCCM guidelines for ICUs in India. Indian journal of critical care medicine : peer-reviewed, official publication of Indian society of critical care medicine. Indian J Crit Care Med..

[19] Rao GS, Aslam M (2021). Inspection plan for COVID-19 patients for Weibull distribution using repetitive sampling under indeterminacy. BMC Med Res Methodol.

[20] Paci A, Borget I, Mercier L, Azar Y, Desmaris RP, Bourget P (2012). Safety and quality assurance of chemotherapeutic preparations in a hospital production unit: acceptance sampling plan and economic impact. J Oncol Pharm Pract.

[21] Wortham AW, Mogg JM (1970). Dependent stage sampling inspection. Int J Prod Res.

[22] Stephens KS, Dodge HF (1976). Comparison of chain sampling plans with single and double sampling plans. J Qual Technol.

[23] Wortham AW, Baker RC (1976). Multiple deferred state sampling inspection. Int J Prod Res.

[24] Balamurali S, Jun CH (2007). Multiple dependent state sampling plans for lot acceptance based on measurement data. Eur J Oper Res.

[25] Aslam M, Yen CH, Chang CH, Jun CH (2014). Multiple dependent state variable sampling plans with process loss consideration. Int J Adv Manuf Technol.

[26] Balamurali S, Aslam M (2019). Determination of multiple dependent state repetitive group sampling plan based on the process capability index. Seq Anal.

[27] Aslam M, Nazir A, Jun CH (2015). A new attribute control chart using multiple dependent state sampling. Trans Inst Meas Control.

[28] Jeyadurga P, Balamurali S (2020). Optimal designing of multiple deferred (dependent) state repetitive group sampling plan for variables inspection. Commun Stat - Theory Methods.

[29] Rao GS, Rosaiah K, Naidu CR (2020). Design of multiple-deferred state sampling plans for exponentiated half logistic distribution. Cogent Math Stat.

[30] Rao GS, Fulment AK, Peter JK (2021). Design of multiple dependent state sampling plan application for COVID-19 data using exponentiated weibull distribution. Complexity.

[31] Aslam M, Jeyadurga P, Balamurali S, Azam M, Al-marshadi A (2021). Economic determination of modified multiple dependent state sampling plan under some lifetime distributions. J Math.

[32] Yen C-H, Chang C-H, Lee C-C (2023). A new multiple dependent state sampling plan based on one-sided process capability indices. Int J Adv Manuf Technol.

[33] Nagasaritha K, Rao GS, Rosaiah K (2023). Survival analysis of cancer patients using a new Lomax Rayleigh distribution. J Appl Math Stat Informatics.

[34] Venegas O, Iriarte YA, Astorga JM, Gómez HW (2019). Lomax-Rayleigh distribution with an application. Appl Math Inf Sci.

[35] Jebeli M, Deiri E (2020). Estimation methods for the probability density function and the cumulative distribution function of the Pareto-Rayleigh distribution. Statistics (Ber).

[36] Al-Anber NJ (2020). Lomax-Rayleigh distribution: traditional and heuristic methods of estimation. J Phys Conf Ser.

[37] Rady EHA, Hassanein WA, Elhaddad TA (2016). The power Lomax distribution with an application to bladder cancer data. Springerplus.

[38] Rana MS, Shahbaz SH, Shahbaz MQ, Rahman MM (2022). Pareto-weibull distribution with properties and applications: a member of Pareto-X family. Pakistan J Stat Oper Res.

[39] Hassan AS, Elgarhy M, Ahmad Z (2019). Type II generalized Topp-Leone family of distributions: properties and applications. J Data Sci.

