# Supplementary material for: Application of the multiple dependent state sampling strategy to late adolescent suicide rates
Source: BMC Med Res Methodol. 2023 Aug 22;23:192. doi: 10.1186/s12874-023-02007-2 (PMC10464250; doi:10.1186/s12874-023-02007-2)
Supplement: Supplementary file 1 — Additional file 1. [file 12874_2023_2007_MOESM1_ESM.docx]

**Appendix**

Data: Worldwide crude suicide rates of 15-19 years per 100000 populations from WHO website.

Data link : who.int/data/gho/data/themes/mental-health/suicide-rates.

6.44,2.43,10.21,2.94,1.25,4.41,3.64,2.41,4.87,4.86,2.99,6.33,4.53,2.59,6.42,4.32,2.66,5.92,4.2,3.55,4.81,2.69,1.75,3.57,4.77,1.81,7.24,1.75,1.08,2.42,5.43,3.79,7.01,14.31,5.94,10.22,0.46,1.66,1.01,13.06,6.64,9.9,3.82,1.13,2.57,3.63,8.59,5.97,4.4,1.19,2.78,4.48,2.02,3.28,6.93,2.21,11.39,3.62,1.95,5.27,6.46,6.85,6.08,6.21,3.06,9.25,2.55,1.43,3.62,9.03,3.8,14.15,6.41,2.32,10.49,8.6,6.28,10.8,5.59,2.44,8.61,5.09,2.73,7.38,2.9,2.22,3.5,4.1,1.71,6.49,3.84,2.31,5.36,2.65,1.2,4.09,2.47,1.19,3.73,5.51,3.76,7.2,3.09,2.79,3.37,4.4,1.93,6.86,8.86,1.38,16.03,3.71,1.89,5.43,1.78,3.42,5.67,4.06,7.21,4.7,2.85,6.41,2.39,1.27,3.31,3.16,2.8,3.51,2.5,1.71,3.27,2.45,2.17,2.72,11.7,9.95,13.38,3.63,3.05,4.16,3.72,1.56,5.8,1.97,1.5,2.41,12.05,8.75,15.19,2.24,1.44,3.02,10.41,7.01,13.64,13.74,13.96,13.54,2.81,1.63,3.95,30.03,20.65,38.85,2.85,0.85,4.84,3.38,1.91,4.78,6,1.79,9.72,2.31,0.74,3.81,4.03,2.61,5.42,2.54,1.23,3.85,3.79,1.84,5.74,3.32,2.1,4.47,1.22,0.72,1.7,10.82,8.55,13,40.37,45.71,35.15,1.28,0.74,1.81,4.4,1.36,7.29,8.09,10.18,6.03,4.4,3.74,5.02,1.98,0.98,2.91,10.4,14.55,6.71,3.26,1.34,5.12,6.87,5.1,8.56,2.83,1.9,3.72,18.89,9.69,27.88,2.42,1.33,3.45,1.85,0.9,2.73,1.1,0.71,1.48,1.16,0.69,1.63,7.84,5.09,10.44,15.35,11.4,19.1,2.24,1.08,3.4,10.04,7.34,12.64,4.28,2.73,5.83,36,19.54,51.93,9.91,10.97,8.93,1.51,0.51,2.35,5.97,4.65,7.26,2.76,2.08,3.42,2.19,1.5,2.87,3.1,2.39,3.78,2.38,0.93,3.82,11.63,11.33,11.94,30.49,16.72,44.23,11.71,4.03,18.90,5.12,6.8,3.54,7.11,1.69,12.36,6.75,4.62,8.79,7.58,3.57,11.33,2.61,1.71,3.52,3.22,1.21,4.85,6.61,4.1,9.06,3.35,2.11,4.51,2.6,1.93,3.25,4.01,4.53,3.52,2.71,1.07,4.34,5.4,3.77,6.9,11.94,8.86,18.94,9.16,5.92,12.44,1.34,0.85,1.83,4.67,2.31,6.95,3.16,1.24,5.08,2.96,1.06,4.75,6.89,2.36,11.47,2.98,1.72,4.19,1.39,0.76,2,6.38,3.8,8.78,4.99,4.11,5.82,5.41,5.5,5.33,9.38,2.78,15.98,10.61,8.19,12.94,6.07,1.67,11.26,10.03,6.22,13.54,3.3,2.47,4.11,2.76,2.74,2.79,1.75,1.5,1.98,2.59,1.59,3.53,5.26,2.6,7.83,4.77,3.43,6.06,1.87,1.2,2.52,8.38,6.76,9.93,4.12,1.42,5.96,4.46,1.87,6.91,14.11,7.26,20.67,2.75,1.78,3.74,1.86,0.86,2.82,3.99,3.2,4.75,2.76,1.37,4.12,9.31,3.94,9.64,17.26,2.15,31.36,2.91,2.46,3.37,11.32,4.8,17.79,4.62,2.11,7.1,2.57,0.76,4.25,2,1.19,2.8,1.14,0.57,1.7,15.25,14.41,16.03,4.02,1.64,6.28,6.88,4.09,9.5,7.11,5.95,8.22,14.76,4.73,24.5,4.11,1.48,6.64,1.78,0.69,2.84,3.16,1.55,4.77,4.37,2.24,6.48,4.4,1.12,7.52,4.18,4.06,4.3,7.8,5.86,9.69,4.47,3.03,5.86,5.07,3.7,6.35,8.28,4.96,11.52,2.58,1.67,3.42,2.63,2.65,2.62,2.53,1.31,3.73,2.67,1.12,4.21,9.39,3.58,14.87,12.21,6.22,17.97,9.42,4.73,13.92,15.38,15.38,15.38,2.48,1.22,3.69,3.66,2.71,4.54,23.88,14.35,32.67,18.13,13.17,22.73,6.24,5.62,6.84,9.18,7.25,11.09,3.44,1.49,5.39,10.37,6.12,15.42.
